# Sponge-Like Water De-/Ad-Sorption versus Solid-State Structural Transformation and Colour-Changing Behavior of an Entangled 3D Composite Supramolecuar Architecture, [Ni_4_(dpe)_4_(btc)_2_(Hbtc)(H_2_O)_9_]·3H_2_O

**DOI:** 10.3390/polym10091014

**Published:** 2018-09-11

**Authors:** Chih-Chieh Wang, Szu-Yu Ke, Kuan-Ting Chen, Ning-Kuei Sun, Wei-Fang Liu, Mei-Lin Ho, Bing-Jyun Lu, Yi-Ting Hsieh, Yu-Chun Chuang, Gene-Hsiang Lee, Shi-Yi Huang, En-Che Yang

**Affiliations:** 1 Department of Chemistry, Soochow University, Taipei 11102, Taiwan; sarah6017@yahoo.com.tw (S.-Y.K.); maxtw3336@gmail.com (K.-T.C.); coco830824@gmail.com (N.-K.S.); yvonne0123weifang@gmail.com (W.-F.L.); jason855180@gmail.com (B.-J.L.); ythsieh@gm.scu.edu.tw (Y.-T.H.); 2 National Synchrotron Radiation Research Center, Hsinchu 30076, Taiwan; chuang.yc@nsrrc.org.tw; 3 Instrumentation Center, National Taiwan University, Taipei 10617, Taiwan; ghlee@ntu.edu.tw; 4 Department of Chemistry, Fu Jen Catholic University, Xinbei 24205, Taiwan; s401192155@gmail.com

**Keywords:** coordination polymer, metal-organic framework, supramolecular architecture, structural transformation, photoluminescence, magnetic property

## Abstract

An entangled composite compound, [Ni_4_(dpe)_4_(btc)_2_(Hbtc)(H_2_O)_9_]·3H_2_O (**1**), where H_3_btc = 1,3,5-benzenetricarboxylic acid and dpe = 1,2-bis(4-pyridyl)ethane, has been synthesized and structurally characterized. Single-crystal structural determination reveals that compound **1** consists of four coordination polymers (CPs), with two two-dimensional (2D) (4,4) layered metal-organic frameworks (MOFs) of [Ni(dpe)(Hbtc)(H_2_O)] and [Ni(dpe)(btc)(H_2_O)]^−^ anion, and two one-dimensional (1D) polymeric chains of [Ni(dpe)(btc)(H_2_O)_3_]^−^ anion and [Ni(dpe)(H_2_O)_4_]^2+^ cation, respectively. The three-dimensional (3D) supramolecular architecture of **1** is constructed via the inter-penetration of inter-digited, double-layered, 2D rectangle-grid MOFs by two 1D coordination polymeric chains, and tightly entangled together via the combination of inter-CPs π–π stacking and hydrogen bonding interactions. The ad-/de-sorption isotherms of 1 for water displays a hysteresis profile with a maximum adsorption of 17.66 water molecules of per molecule unit at relative P/P_0_ < 0.89. The reversible de-/re-hydration processes in **1** monitored by cyclic water de-/ad-sorption TG analysis and PXRD measurements evidence a sponge-like water de-/ad-sorption property associated with a thermal-induced solid-state structural transformation. The magnetic property of **1** suggests that the ferromagnetic coupling might refer to a stronger inter-Ni(II) interaction, which could be along the btc^3−^ or Hbtc^2−^ ligands; the antiferromagnetic coupling corresponding to the weaker inter-Ni(II) interactions, which could be the dpe ligands for the 2D framework.

## 1. Introduction

The study of 3D supramolecular architectures assembled via coordination polymers (CPs) [1] or metal organic frameworks (MOFs) [1,2] with various types of structural topologies is an important research topic, not only in terms of their structural diversity in the solid-state structural studies of supramolecular chemistry [3,4,5,6,7,8,9,10,11,12], but also for their potential functional applications. A variety of supramolecular architectures have been obtained on the concepts of crystal engineering assembled by a large range of bonding forces, ranging from the classic M–L covalent bonds, to strong halogen interactions [13,14,15,16,17,18,19,20,21] or hydrogen bonds [22], to much weaker forces, such as weak hydrogen bonds [23,24] and π–π stacking interactions of small aromatics [25,26,27,28]. In the field of supramolecular chemistry, entangled 3D supramolecular networks serve as an important subject via the assembly of various CPs, as seen in interpenetration, polycatenane, interdigitation, polythread, and other species [29,30,31,32,33,34,35,36]. Two or more CPs with different dimensionality—such as 1D plus 1D, 1D plus 2D, 2D plus 2D, and 2D plus 3D—within the crystal were found in the formation of their appealing interpenetrated or catenated 3D supramolecular networks [37,38,39,40,41,42,43,44,45,46,47,48]. Recently, a entangled 3D architecture composed of four CPs in a Co_4_ system with chemical formula [Co_4_(dpe)_4_(BTC)_2_(HBTC)(H_2_O)_8.5_(EtOH)_0.5_]·3H_2_O (dpe = 1,2-bis(4-pyridyl)ethane and H_3_BTC = 1,3,5-benzenetricarboxylic acid) has been reported in our groups, which shows a composite combination of two 1D chains plus two 2D layers, extending to a 3D entangled supramolecular network [49]. Benzene-1,3,5-tricarboxylic acid (H_3_btc) is a rigid, planar molecule, and has been widely used as a bridging ligands on the construction of multi-dimensional MOFs in the form of its three benzene-1,3,5-triboxylate anions, H_2_btc^−^, Hbtc^2−^, and btc^3−^ [50,51,52,53,54,55,56,57,58,59,60,61,62,63,64,65,66,67,68,69,70,71,72,73,74,75,76,77,78,79,80,81,82,83,84,85,86,87,88,89,90,91]. Among these polymeric frameworks, the mixed-ligands Ni(II) CPs with 2D or 3D polymeric frameworks consisting of [Ni(Hbtc)] [54,55,56,57,58,59,60] or [Ni_3_(btc)_2_] [68,71,72,73,74,75,76,77,78,79,80,81,82,83] moieties as building units associated with nitrogen-based co-ligands have been investigated, and shown to possess interesting physical properties. However, composite 3D supramolecular architectures composed of two or more CPs are rare; only one example of [Ni(H_2_biim)_3_][Ni(btc)(Hbim)] 2H_2_O (H_2_biim = 2,2′-biimidazole) is reported with its unique hydrogen-bonded pillared-layer 3D network being assembled by a 0D monomeric cation, [Ni(H_2_biim)_3_]^2+^, and a 2D anionic layers, [Ni(btc)(Hbim)]^2−^ [68]. In this work, we report on the exploration of a composite “four in one” supramolecular network, [Ni_4_(dpe)_4_(btc)_2_(Hbtc)(H_2_O)_9_]·3H_2_O (**1**), with [Ni(Hbtc)] and [Ni(btc)] moieties obtained by the reaction of Ni(II) nitrate with dpe and H_3_btc ligands. This example represents a 3D supramolecular architecture composed of four crystallographiclly independent CPs, that is, two 2D (4,4) layered CPs, [Ni(dpe)(Hbtc)(H_2_O)] and [Ni(dpe)(btc)(H_2_O)]^−^, and two 1D linear chain-like CPs, [Ni(dpe)(btc)(H_2_O)_3_]^−^ and [Ni(dpe)_2_(H_2_O)_4_]^2+^, which are entangled together via the inter-penetration of the rectangular channels of inter-digitated 2D double-layered frameworks by 1D polymeric linear chains. The de-hydrated species **1**, after controlled heating at 240 °C, shows remarkable thermal-induced colour-changing behavior and reversibility to yield a re-hydrated structure when exposed to water vapor at 30 °C, indicating a sponge-like water de-/ad-sorption behavior and solid-state structural transformation.

## 2. Materials and Methods

### 2.1. Materials and Physical Techniques

General: All chemicals were of reagent grade and were used as commercially obtained without further purification. Elementary analyses (carbon, hydrogen and nitrogen) were performed using a Perkin-Elmer 2400 elemental analyzer (PerkinElmer, Taipei, Taiwan). IR spectra were recorded on a Nicolet Fourier Transform IR MAGNA-IR 500 spectrometer (Thermo Fisher Scientific, Waltham, MA, USA) in the range of 500–4000 cm^−1^ using the KBr disc technique. Diffuse reflectance UV-vis spectra of **1** were obtained with a HITACHI U-4100 spectrophotometer equipped with an integrating sphere accessory (Al_2_O_3_ was used as a reference). Thermogravimetric analysis (TGA) of compound **1** was performed on a computer-controlled Perkin-Elmer 7 Series/UNIX TGA7 analyzer (PerkinElmer, Taipei, Taiwan). The adsorption isotherm of H_2_O (298 K) was measured in the gaseous state by using BELSORP-max volumetric adsorption equipment from BEL, Osaka, Japan. In the sample cell (~1.8 cm^3^) maintained at *T* ± 0.03 K was placed the adsorbent sample (~100–150 mg), which has been prepared at 180 °C for **1** and 10^−2^ Pa for about 24 h prior to measurement of the isotherm. The adsorbate was placed into the sample cell; the change of pressure was then monitored, and the degree of adsorption was determined by the decrease of pressure at equilibrium state. All operations were automatically computer-controlled. Single-phased powder samples were loaded into alumina pans and heated with a ramp rate of 5 °C/min from room temperature to 800 °C under a nitrogen atmosphere. Diffuse reflectance UV-vis spectra of **1** were obtained with a HITACHI U-3900Hspectrophotometer (Hitachi High Technologies America, Inc., Schaumburg, IL, USA) equipped with an integrating sphere accessory (Al_2_O_3_ was used as a reference). The temperature-dependent magnetic susceptibility was measured on the SQUID system with 2 kOe external magnetic field.

### 2.2. Synthesis of [Ni_4_(dpe)_4_(btc)_2_(Hbtc)(H_2_O)_9_] 3H_2_O *(**1**)*

A solution of 1,3,5-benzenetricarboxylic acid (H_3_btc, 4.2 mg, 0.02 mmol) and 1,2-bis(4-pyridyl)ethane (dpe, 5.2 mg, 0.03 mmol) in mixed solvents of distilled water and EtOH (1:1, *v*/*v*) (9 mL) was added to a solution of Ni(NO_3_)_2_·6H_2_O (8.72 mg, 0.03 mmol) in mixed solvents of distilled water and EtOH (1:1, *v*/*v*) (3 mL) to give a pale-green solution which was left to stand at 80 °C in the oven for 72 h, and then at RT for several days. Light-blue plate-shaped crystals were collected by filtration and washed several times with distilled water, and then dried in air; yields of 40.3% were obtained. Elemental Analysis Calculated (Anal. Calc.) or C_75_H_82_N_8_Ni_4_O_30_ (*M*_w_ = 1810.33): C 49.76, N 6.19, H 4.57. Found: C 49.90, N 6.15, H 4.23. IR (KBr pellet): ν = 3468 (w), 3461 (w), 3037 (w), 1698 (s), 1635 (m), 1618 (m), 1559 (vs), 1427 (m), 1236 (s), 766 (m) cm^–1^.

### 2.3. Crystallographic Data Collection and Refinements

Single-crystal structural analysis for compound **1** was performed on a Siemens SMART diffractometer with a CCD detector with Mo radiation (λ = 0.71073 Å) at 100 K. A preliminary orientation matrix and unit cell parameters were determined from 3 runs of 15 frames each, with each frame corresponding to a 0.3° scan over 10 s, followed by spot integration and least-squares refinement. For each structure, data were measured using ω scans of 0.3° per frame for 20 s until a complete hemisphere had been collected. Cell parameters were retrieved using SMART [92] software and refined with SAINT [93] on all of the observed reflections. Data reduction was performed with the SAINT [94] software and corrected for Lorentz and polarization effects. Absorption corrections were applied with the program SADABS [94]. Direct phase determination and subsequent difference Fourier map synthesis yielded the positions of all non-hydrogen atoms, which were subjected to anisotropic refinements. All hydrogen atoms were generated geometrically (C–H = 0.93 (C_sp__2_–H) or 0.97 (C_sp__3_–H) Å), with the exception of those of the coordinated water molecules, which were located in the difference Fourier map with the corresponding positions and isotropic displacement parameters being refined. The final full-matrix, least-squares refinement on *F*^2^ was applied for all of the observed reflections (I > 2σ(I)). All calculations were performed using the SHELXTL-PC V 5.03 software package (Siemens Analytical Instruments Division, Madison, WI, USA) [95]. Crystal data and details of the data collection and structure refinements for **1** are summarized in Table 1. CCDC-1498095 for **1** contains the supplementary crystallographic data for this paper. These data can be obtained free of charge at www.ccdc.cam.ac.uk/conts/retrieving.html or from the Cambridge Crystallographic Data Centre, 12, Union Road, Cambridge CB2 1EZ, UK; fax: (internat.) +44-1223/336-033; email: deposit@ccdc.cam.ac.uk.

### 2.4. In Situ X-ray Powder Diffraction

The synchrotron powder X-ray diffraction data of **1** were collected at the BL01C2 beamline at the National Synchrotron Radiation Research Center (NSRRC) in Taiwan. The wavelength of the incident X-rays is 1.03321 Å and the diffraction patterns were recorded with a Mar345 imaging plate detector placed approximately 331 mm from sample positions. The one-dimensional powder diffraction profile was converted with program FIT2D [96] and cake-type integration, where the diffraction angles were calibrated according to Bragg positions of Ag-Benhenate and Si powder (SRM640c) standards. In-situ temperature dependent experiment for **1** was performed from 28 to 300 °C, with a heating rate 10 °C/min. The powder sample was packed in a glass capillary (0.3 mm diameter) and heated in a stream of hot air; each pattern was exposed for about 1.2 min. The capillary of de-hydrated sample was immersed in water for one hour, then the powder pattern was measured again.

## 3. Results and Discussion

### 3.1. Synthesis and Structural Description of [Ni_4_(dpe)_4_(btc)_2_(Hbtc)(H_2_O)_9_]·3H_2_O *(**1**)*

**Scheme 1:**
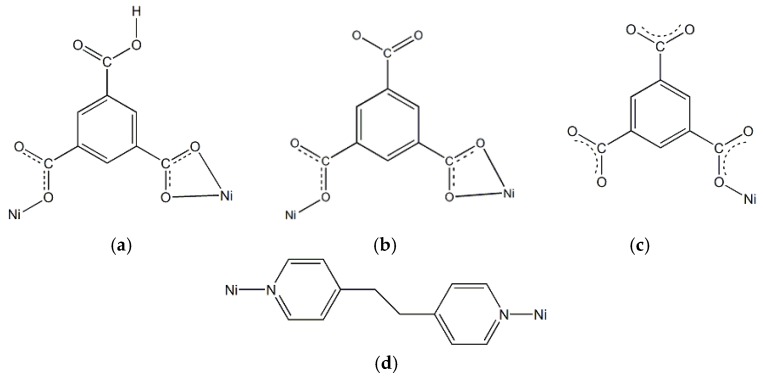
(**a**) chelating/monodentate coordination mode of Hbtc^2−^ in **A**; (**b**) chelating/monodentate coordination mode of btc^3−^ in **B**; (**c**) monodentate coordination mode of btc^3−^ in **C**; and (**d**) *bis*-monodentate coordination mode of dpe.

**Figure 1:**
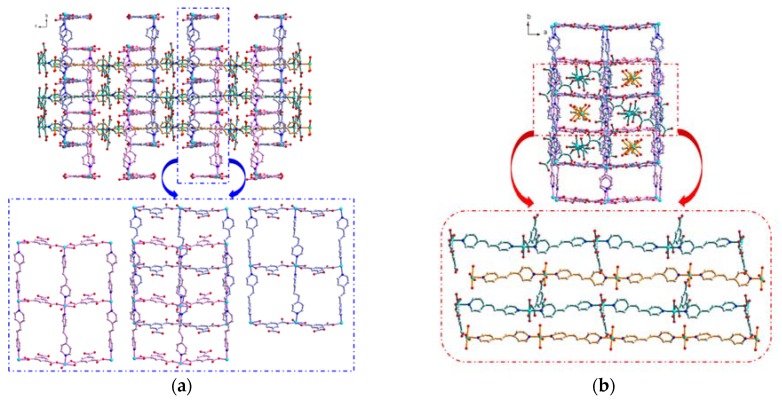

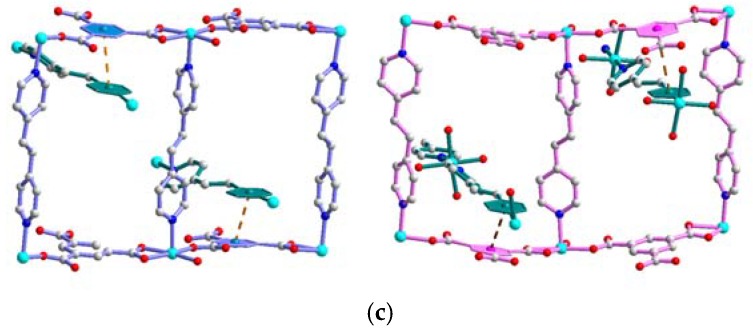
(**a**) Up: 3D supramolecular entangled architecture in **1** showing the interpenetration of two 1D chains (**C** and **D**) into the rectangular channels formed by 2D double-layered network (**A** plus **B**) viewing along the *a* axis. Bottom, middle: 2D double-layers of A plus B viewing along the *c* axis; right: 2D rectangle-grid layer of A, with grid-dimensions of 10.263 × 13.668 Å; left: 2D rectangle-grid layer of B, with grid-dimensions of 10.184 × 13.668 Å, (**b**) up: 3D supramolecular entangled architecture in 1 viewing along the *c* axis showing the interpenetration of two 1D chains (**C** and **D**) in an alternate array. Bottom: two 1D linear chains of **C**, and **D**, (**c**) π–π interactions between the pyridine rings of *anti*-dpe ligands and the benzene rings of the Hbtc^2−^ or btc^3−^ ligands.

Compound **1** was synthesized by the reactions of the mixing of nickel(II) nitrate, dpe, and H_3_btc in ethanol/water solution with molar ratios of 3:3:2 standing in the oven at 80 °C, resulting in the formation of light-blue crystals of **1**, formulated as [Ni_4_(dpe)_4_(btc)_2_(Hbtc)(H_2_O)_9_]·3H_2_O, which are suitable for X-ray diffraction analysis. Structural determination reveals that the crystal structure of **1** show the presence of four crystallographically independent polymeric structures entangled together: containing two 2D layered CPs of neutral [Ni(dpe)(Hbtc)(H_2_O)] **A**, anionic [Ni(dpe)(btc)(H_2_O)]^−^ **B**, two 1D polymeric chain-like CPs of anionic [Ni(dpe)(btc)(H_2_O)_3_]^−^ **C**, and cationic [Ni(dpe)(H_2_O)_4_]^2+^ **D**, respectively. In total, **1** can be formulated as [Ni(dpe)(Hbtc)(H_2_O)][Ni(dpe)(btc)(H_2_O)][Ni(dpe)(btc)(H_2_O)_3_][Ni(dpe)(H_2_O)_4_]]·3H_2_O; all the oxidation states of four independent nickel centers are 2+. The coordination environments of Ni(II) ions in **A** and **B** are both six-coordinate with a distorted octahedral geometry, (shown in Figure S1a,b in the supplementary materials, respectively), bonded to two nitrogen atoms of two *anti*-dpe, and four oxygen atoms of one water molecule and two Hbtc^2−^ ligands in **A**, and two btc^3−^ ligands in **B**, respectively. The related bond-lengths around the Ni(II) ions are listed in Table S1 (Supplementary Materials). The Hbtc^2−^ in **A** and btc^3−^ in **B** both act as the bridge ligands with chelating/monodentate coordination mode (Scheme 1a,b) connecting the Ni(II) ions to form linear chains. Adjacent chains are then mutually connected via the bridges between the Ni(II) ions and dpe ligand with *bis*-monodentate coordination mode (Scheme 1d), to generate 2D layered MOFs (Figure 1a) with a 4^4^ structural topology by using rectangle-grid as the basic building block. The rectangle-grid dimensions are 10.263 × 13.668 Å (Figure 1a, bottom, left) via the bridges of Hbtc^2−^ and *anti*-dpe in **A**, and 10.184 × 13.668 Å (Figure 1a, bottom, right) via the bridges of btc^3−^ and *anti*-dpe in **B**, respectively. Moreover, **A** and **B** are mutually inter-digitated into each other along the direction of the uncoordinated carboxylate groups of the lateral Hbtc^2−^ and btc^3−^ ligands, respectively, to fabricate a 2D double-layered {**AB**} network (Figure 1a, bottom, middle), resulting in the formation of inner rectangular channels with dimensions of ca. 6.834 × 10.223 Å. The lateral Hbtc^2−^ in **A** and btc^3−^ in **B** are oriented vertically up and down, respectively, into the double-layered network as the walls of the channels. The coordination environments of Ni(II) ions in **C** and **D** are both six-coordinate (shown in Figure S1c,d deposited in the supplementary materials), in which the Ni(II) ion in **C** has a distorted {NiN_2_O_4_} octahedral geometry (Figure S1c, Supplementary Materials) which is bonded to two nitrogen atoms of two *anti*-dpe ligands with *bis*-monodentate coordination mode (Scheme 1d, Supplementary Materials), four oxygen atoms of one btc^3−^ ligand with monodentate coordination mode (Scheme 1c, Supplementary Materials), and three water molecules, while the Ni(II) ion in **D** also has a distorted {NiN_2_O_4_} octahedral geometry (Figure S1d, Supplementary Materials) bonded to two nitrogen atoms of two *anti*-dpe ligands with *bis*-monodentate coordination mode (Scheme 1d, Supplementary Materials), and four oxygen atoms of four water molecules. The related bond lengths around the Ni(II) ions are listed in Table S1. Both **C** and **D** are 1D polymeric linear chains via the connectivity between the Ni(II) ions, and *anti*-dpe ligands extending along the *c* axis, as shown in Figure 1b.

**Figure 2:**
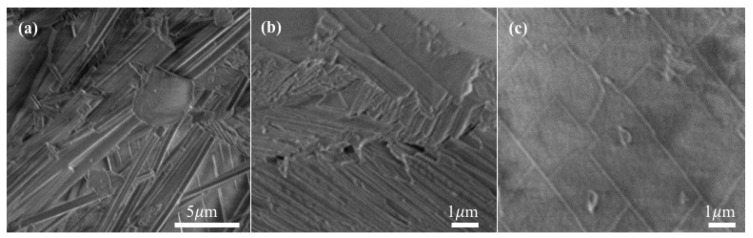
SEM images of crystals **1** with different magnifications (**a**) the global view; (**b**,**c**) enlarged images at different positions. The acceleration voltage is 2 kV.

The most remarkable and interesting structural feature of **1** is the 3D tightly-entangled supramolecular architecture, which is composed of a 2D **{**[Ni(dpe)(Hbtc)(H_2_O)]^−^[Ni(dpe)(btc)(H_2_O)]**}**, ({**AB}**) double-layers and two 1D [Ni(dpe)(btc)(H_2_O)_3_]^−^ (**C**) and [Ni(dpe)_2_(H_2_O)_4_]^2+^ (**D**) polymeric chains, as shown in Figure 1a,b. First of all, adjacent double-layered frameworks of {A plus B} are arranged parallel in an {**AB**}{**BA**}{**AB**}{**BA**}…manner (Figure 1a, up) along the *c* axis, generating 1D rectangular channels (Figure 1b, up). These channels are then fully occupied by the 1D polymeric linear chains of **C** and **D** in an alternate **CDCD** sequence to complete its unique 3D supramolecular array. To the best of our knowledge, this is the first 3D entangled Ni_4_ supramolecular network constructed via the composite combination of two 1D chain-like CPs plus two 2D layered CPs by inter-digitation and inter-penetration. Most importantly, the subtle combination of hydrogen-bonding and π…π stacking interactions among the four CPs both play significant roles in the construction of its 3D tightly entangled architecture. Firstly, 4 sets of intra-CP O–H…O type hydrogen bonds with the O…O distances in the ranges of 2.518(9)–2.861(9) Å, and 11 sets of inter-CPs O–H…O type hydrogen bonds with the O…O distances in the ranges of 2.591(9)–2.830(9) Å between the coordinated water molecules and the oxygen atoms of Hbtc^2−^ or btc^3−^ ligands among the four CPs, provide extra energy on the stabilization of the entangled architecture. Furthermore, three solvated water molecules intercalated in the vacant pores of the 3D supramolecular architecture are reinforced by 9 sets of intermolecular O–H…O type hydrogen bonding interactions between the solvated water molecules and the oxygen atoms of coordinated water molecules, Hbtc^2−^ and btc^3−^ ligands, with O…O distances in the range of 2.643–2.941 Å. The mutually linkage of intra-CP and inter-CP hydrogen bonding interactions among four CPs and solvated water molecules not only stabilize the entangled 3D network, but also provide the possible pathways for the reversible water de-/ad-sorption behavior during thermally de-/re-hydration processes, which will be discussed in the following section. The common hydrogen-bonding distances and angles in **1** are summarized in Table S2 (Supplementary Materials). Furthermore, π–π stacking interactions between the pyridine rings of *anti*-dpe ligands and the benzene rings of the Hbtc^2−^ or btc^3−^ ligands, with the ring centroid distances in the range of 3.498–3.995 Å, provide the extra stabilization energy on the construction of the 3D entangled assembly of **1**. A schematic representation of two π–π interactions with shorter ring centroid distances is shown in Figure 1c. The related inter-planar parameters are listed in Table S3 (deposited in Supplementary Materials). The surface morphology of the crystals of **1**, through the SEM images in Figure 2a, shows rod-shaped formations. The enlarged images at different positions (Figure 2b,c) reveal that **1** was stacked up with numerous crack-free, 2D layers; each layer is completely made up of small crystals.

### 3.2. Thermal Stability of ***1*** by TG Analysis and In Situ Powder X-ray Diffraction Analyses

**Figure 3:**
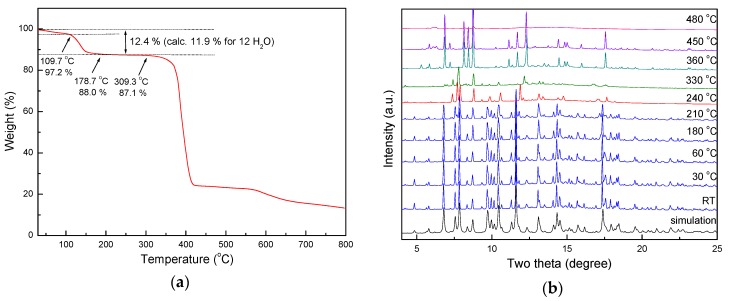
(**a**) Thermogravimetric (TG) analysis of **1**; (**b**) In-situ PXRD patterns of **1** at different temperatures, and simulated PXRD pattern from single-crystal X-ray diffraction data.

A thermogravimetric analysis (TGA) of **1** was performed to assess the thermal stability on the 3D entangled network as a function of temperature. During the heating process, the TG analysis (Figure 3a) revealed that **1** underwent a two-steps weight loss of total 12.4%, which corresponded to the loss of 3 solvated water molecules (calc 3.0%), occurring in the range of approximately 31–109 °C for the first step, and 9 coordinated water molecules (calc 9.0%), occurring in the range of approximately 109–178 °C for the second step. In the temperature range of approximately 178–310 °C, de-hydrated **1** was stable without any weight loss. On further heating, these samples decomposed at approximate 310 °C. In order to gain structural variation in depth upon de-hydration of water molecules, in situ synchrotron X-ray powder diffraction patterns of **1** were collected continuously from 25 to 480 °C; the results at some specific temperatures are shown in Figure 3b. The powder pattern of fresh sample at RT matches well with the simulated pattern based on the single crystal structure. As the temperature increases, a phase transition occurred at 210 °C, and converted to a meta-stable phase at 240 °C. It is unfortunate that, due to the poor long range ordering of PXRD data, the unit cell of de-hydrated form **1** could not be determined directly using synchrotron PXRD diffraction data.

### 3.3. *Reversible* Water De-/Ad-Sorption Behavior Accompanying with Morphological Changes during the Thermal De/Re-Hydration Processes

**Figure 4:**
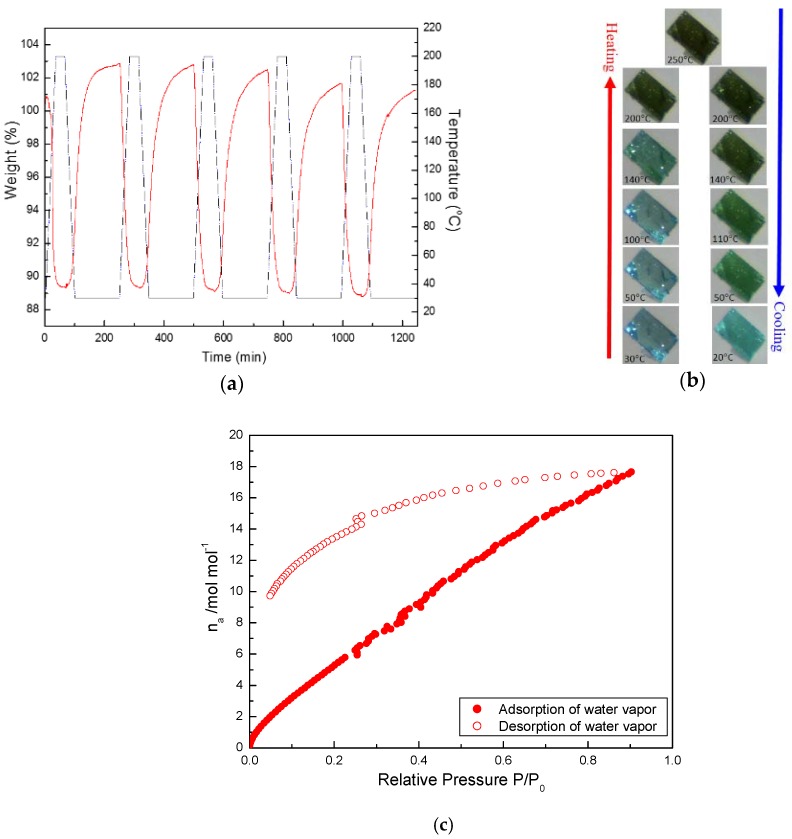
(**a**) TG measurements of cyclic de-/re-hydration processes for **1** were repeated four times. Solid red line: the variation of weight loss with time; black dash-dot line: the variation of temperature with time. (**b**) Photographs of plate-shaped crystal **1** at different temperatures. (**c**) Water Vapor ad-/de-sorption isotherms at 298 K for **1**.

In order to verify the reversibility of water de-/ad-sorption property of **1** during the re-/de-hydration processes, cyclic TG measurements were taken under water vapor by thermal treatment, as shown in Figure 4a. The de-hydrated **1** after the de-hydration process by the thermal treatment (up to 200 °C) shows a 10.7% weight loss, corresponding to 10.8 H_2_O molecules. The water molecules can then be re-adsorbed by exposing the de-hydrated **1** to water vapor, forming a re-hydrated crystal with a weight increase of 13.4%, corresponding to approximate 13.5 water molecules when the sample cooled down to room temperature. Such heating (up to 200 °C) and cooling (down to RT) procedures were repeated for five cycles, as shown in Figure 4a, with almost the same weight-increase/weight-decrease percentages (12.4%~13.5%); this was in order to demonstrate the stable reversibility of the thermal re-/de-hydration processes. This result evidences that **1** adopts a reversible water de-/ad-sorption sponge-like property between de-hydrated and re-hydrated forms, driven by thermal re-/de-hydration treatments. It is also important to note that this water de-/ad-sorption sponge-like property was accompanied by morphological changes. The simultaneous and gradual colour change of the crystal during the de-/re-hydration processes was followed by optical microscopy (Figure 4b). A Well-formed blue crystal of **1** was obtained as a plate-shaped sheet, shown in Figure 4b (left, bottom), at RT. **1** undergoes reversible colour-changing behavior when the temperature rises, with the colour gradually shifting from blue to brown-green (Figure 4b, left side from bottom to top). As the temperature was raised from RT to 50, 100, 140, 200 and 250 °C, the colour of dried crystal gradually became deeper than that at RT, and many chaps with random cracks on the crystal surface were observed. The obvious colour change from blue to brown-green took place from 140 to 200 °C upon the loss of solvated and coordinated water molecules, respectively. Furthermore, the brown-green de-hydrated crystal was slowly turned back to a blue, re-hydrated one (Figure 4b, right side from top to bottom) as the temperature decreased from 250 °C to RT, indicating the slow re-hydration procedure from water vapor in the air. Finally, the brown-green de-solvated sample gradually returned to its original blue as the crystal was exposed to air at RT and left to stand for more than one day. However, the transparency of the re-hydrated crystal was not as high as that of the fresh crystal. In order to understand the interactions of guest and coordinated water molecules within the host framework, the water vapor sorption performance of de-hydrated **1** was also studied at 298 K (Figure 4c). For water vapor adsorption, an increase in the amount of adsorbed vapor was found at 0 < relative *P*/*P*_0_ < 0.89, with maximum adsorption of 17.66 water molecules of per molecule unit at relative *P*/*P*_0_ < 0.89, indicating that nine coordinated water molecules, and more than three guest water molecules (found in the crystal structure) were absorbed. Such an observation is in agreement with the result in the cyclic TG measurements (Figure 4a). It is worth noting that the desorption curve did not trace the adsorption curve any longer, which exhibited a large hysteresis with the 9.73 water molecules of per molecule unit at relative *P*/*P*_0_ = 0.05, nearly corresponding to 9 coordinated water molecules found in the crystal structure. This result reveals that the water molecules are strongly interacted with the 1D or 2D CPs, not only through the coordination with Ni(II) ions, but also through hydrogen bonding interactions; therefore, desorption was rendered difficult, resulting in a hysteresis profile. The water adsorption had dynamic adsorption property, wherein the de-hydrated host framework changed according to the water molecules entering. Further study of the sponge-like water de-/adsorption property correlated with the thermal-induced solid-state structural transformation and UV-vis absorption properties. Other properties of **1** will be discussed in the next sections.

### 3.4. Solid-State Structural Transformation by Thermal De-/Re-Hydration Processes

**Figure 5:**
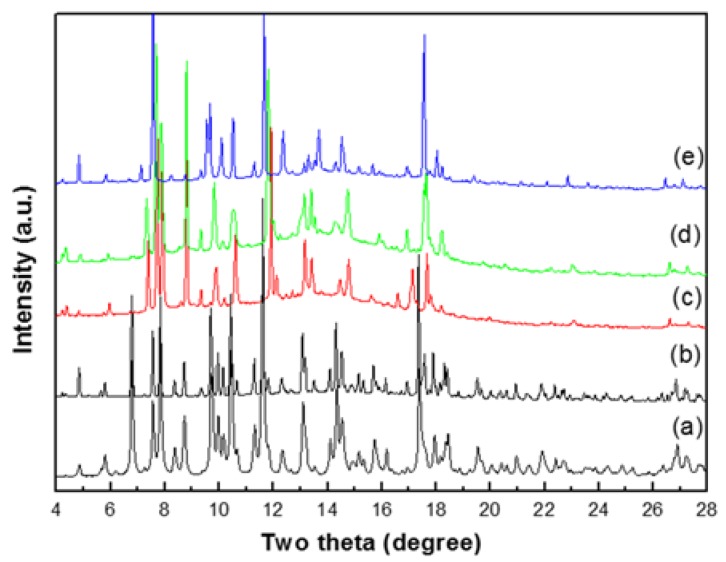
PXRD patterns of (**a**) simulation of **1** from single-crystal X-ray diffraction data; (**b**) fresh samples of **1** at RT; (**c**) De-hydrated samples of **1** at 240 °C; (**d**) the de-hydrated samples **1** exposed to the air at RT; (**e**) the re-hydrated samples obtained by the de-hydrated samples **1** immersed in the water solution.

**Figure 6:**
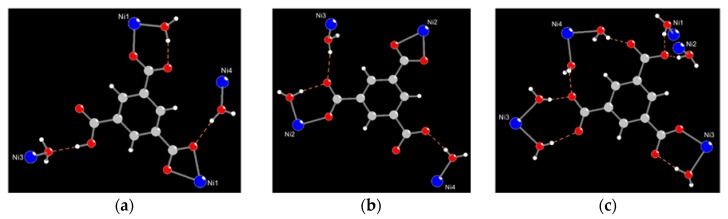
The proximity of intra- and inter-molecular hydrogen bonds between the carboxylate fragments of (**a**) Hbtc^2−^ fragment in **A**; (**b**) btc^3−^ fragment in **B**; (**c**) btc^3−^ fragment in **C** respectively, and the coordinated water molecules.

The most promising feature of crystal **1** is that it adopts sponge-like reversible water de-/ad-sorption behavior by thermal de-/re-hydration processes, which has been identified by cyclic TG and morphological measurements. Furthermore, the correlation between the water de-/ad-sorption sponge-like property and the dynamic solid-state structural transformation during the de-/re-hydration processes is also important, and worthy of further study by PXRD analysis. As shown in Figure 5c,e, when the de-hydrated samples of **1** at 240 °C is cooled down to RT and exposed to water (i.e., the de-hydrated sample is placed in a glass capillary, beside a beaker filled with water or immersed in the beaker), it re-absorbs the water molecules. The PXRD pattern of the re-hydrated species that immersed into the water solution (Figure 5e) is not matched so well to that of the freshly synthesized samples (Figure 5b), which indicates that the structure of re-hydrated species may be slightly different or incompletely transferred to the original structure **1**, and is re-established as a new re-hydrated form. The results indicates that **1** may undergoes a reversible dynamic solid-state structural transformation between the de-hydrated form and a new re-hydrated form, and that the re-hydrated structure of **1** may be similar but slightly different to its original structure, which has been demonstrated from the water ad-/de-sorption isotherms (shown in Figure 4c), with more than three guest water molecules being absorbed. Owing to the structures of the de-hydrated form in the high temperature phase, and rehydrated species at RT, the structures cannot be determined directly by PXRD data; therefore, the structural transformation mechanism for the thermal de-hydration processes is not clear. However, the reversibly dynamic solid-state structural transformation may be attributed to the significant hydrogen bonding interactions among the coordinated, solvated water molecules and oxygen atoms of the Hbtc^2−^ and btc^3−^ ligands in four CPs. The relatively easy release of the coordinated water molecules under a gentle thermal process is indeed made possible by a carboxylate-assisted process; a structural drawing illustrating the proximity of carboxylate fragments (Hbtc^2−^ and btc^3−^) and the coordinated water molecules among four CPs is shown in Figure 6. A possible route for the reversible structural transformation route between de-hydrated form and re-hydrated form could be that the removal of the coordinated water molecules creates open sites of Ni(II) centers for the approach of neighboring oxygen atoms of the Hbtc^2−^ and btc^3−^ ligands, which are also oriented towards the coordination water molecules of neighboring units via O–H…O hydrogen bonds [97]. These contacts shorten in the course of the solid-state reaction, and turn into bonds with Ni(II) atoms after the removal of the water molecules to generate the de-hydrated form, and then re-absorb to form the re-hydrated form after the de-hydrated sample is exposed to water vapor.

### 3.5. Colour-Changing Behavior and *UV-vis Absorption* Property of De-/Re-Hydration for Crystal ***1***

**Figure 7:**
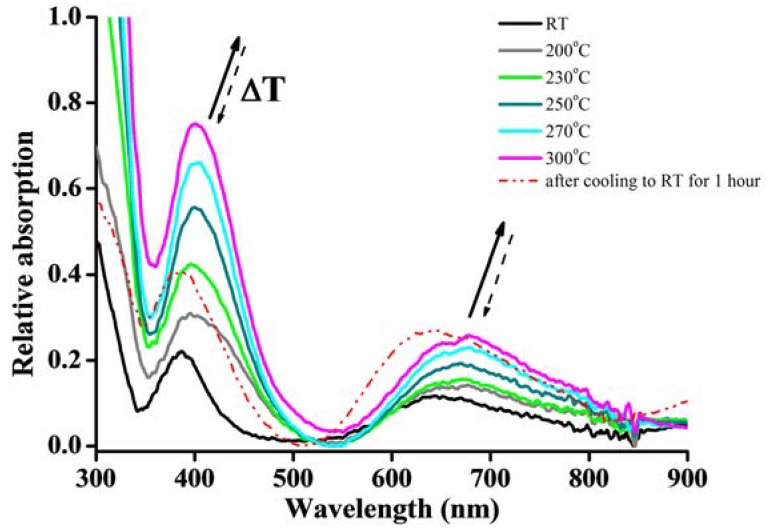
The UV-vis diffuse reflectance spectra of **1** upon heating and cooling from RT to 300 °C.

Finally, as shown in Figure 7, the crystal **1** shows two bands at 387 and 645 nm, which may be attributed to the ^3^A_2g_(F) → ^3^T_1g_(P) and ^3^A_2g_(F) → ^3^T_1g_(F) electronic transitions, respectively; this indicates an octahedral geometry around the Ni(II) center [98,99,100]. These transitions are consistent with the crystal structure of **1**. To further observe the temperature-dependent change processes of the crystal, in situ heating/cooling UV-vis absorption spectrascopy was used to characterize the change of these two transitions. As depicted in Figure 7, upon heating from RT to 300 °C, the intensity of these two bands tends to increase, accompanied by the gradual red-shift of the bands. This result indicates that the coordination environments around the Ni(II) have been changed upon heating, and hence, result in the energy change of d-d transitions. In particular, after heating to 170 °C, the absorbance at 387 nm had a maximum shift with 8 nm to 395 nm, which can be attributed to the loss of the coordination waters around the Ni(II). This result is in accordance with that of the TG and PXRD analyses (vide supra). Furthermore, as the temperature decreased from 200 °C to RT after one hour, the absorption shifted back to the 387 nm (see dash line), which is the same position at RT, and accompanied the intensity enhancement. This indicates that the water has come back to the coordination center during the rehydration process. The rehydration crystal at RT has a more intense absorption intensity than that of the original crystal, which may be attributed to the fact that the rehydration structure is slightly different compared to that of **1** (vide supra), and hence, leads to a different d-orbital energy state change.

### 3.6. Magnetic Property of ***1***

**Figure 8:**
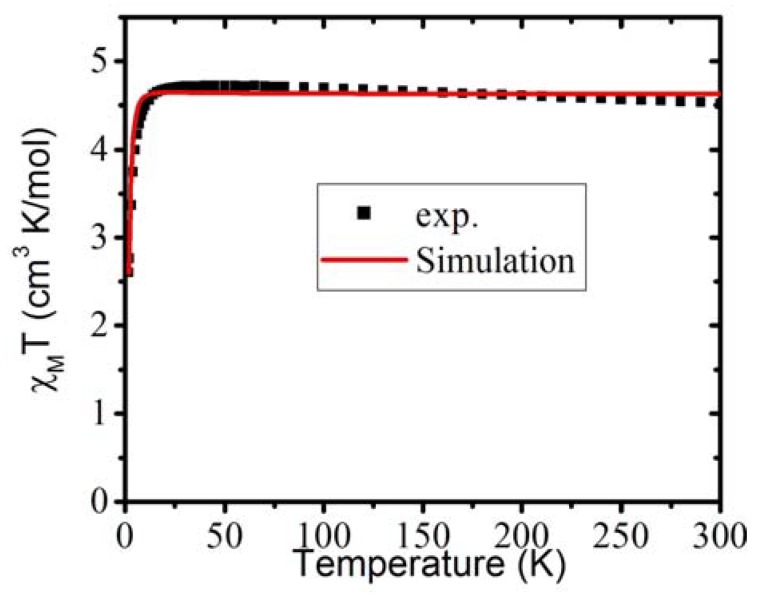
The magnetic χ_M_T vs. T plot of compound **1**, where the black squares are the experimental data, and the simulation based on Equation (1) is the red solid line.

The magnetic properties of compound **1** were explored by dc magnetic susceptibility measurements. The experiment was performed on a polycrystalline sample under a 2000 G magnetic field in the temperature range of 2 to 300 K. Figure 8 illustrates the χ_M_T vs. T plot, with the experimental data as the black square points. It can be seen that the χ_M_T value is about 4.5 cm^3^ K/mol at 300 K, which corresponds to four uncoupled Ni(II) ions with *g* = 2.13. Upon cooling, the χ_M_T values slightly go up and reach the maximum 4.72 cm^3^ K/mol at 45 K. The behavior of the increase of the χ_M_T values on cooling indicates a weak ferromagnetic coupling dominant on one direction of the 2-dimensional net structure. With continued cooling, the χ_M_T values slowly decrease to 4.0 cm^3^ K/mol at 5 K, and drop abruptly to 2.76 cm^3^ K/mol at 2 K. This behavior strongly suggests that an antiferromagnetic coupling interaction is in the other direction of the 2-dimensional network. To simulate this majorly 2-dimensional network of Ni(II) ions, a 2-D Ising-like coupling scheme was employed.

H = −∑ 2*J*_1_ S(i,j)·S(I + 1,j) − ∑ 2*J*_2_ S(i,j)·S(i,j + 1) (1)

The simulation was done by applying Equation (1) to the program MAGPACK [101,102]. The result of the simulation, based on Equation (1), is presented as red solid line in Figure 8, which gives *J*_1_ = +0.4 K, *J*_2_ = −0.34 K and *g* = 2.15. It is clear that the strength of ferromagnetic coupling is stronger than the antiferromagnetic coupling, so that the χ_M_T values increase on cooling when the temperatures are high, but drop deeply at very low temperatures (~2 K). Judging from the structure, there seems to be two pathways that can conduct magnetic coupling, i.e., btc^3−^/Hbtc^2−^ and dpe ligands. From the literature, most btc ligand binding Ni(II) ions are antiferromagnetic coupling [103,104], whereas the dpe ligand, due to its large size, usually ignores the magnetic exchange coupling [105]. As the ferromagnetic coupling, in our case, is stronger than the antiferromagnetic one, it seems to be unreasonable to suggest that the ferromagnetic coupling came purely from the dpe pathway. Therefore, we suggest the antiferromagnetic coupling may be the result of btc^3−^/Hbtc^2−^ bridging; however, the source of the ferromagnetic coupling still remains unclear.

## 4. Conclusions

In conclusion, compound **1** can be considered as a “four-in-one” nonporous, supramolecular network, with its 3D entangled architecture being constructed via the inter-digitation of two crystallographically independent CPs forming porous (2D plus 2D) interdigitated double-layers, and then via the inter-penetration by two 1D crystallographically independent chain-like CPs, which displays an interesting moisture-sensitive induced colouring-changing property. Intra-CP and inter-CPs hydrogen bonding interaction and π–π interactions among the four CPs play key roles in the stabilization of the 3D entangled architecture, showing high thermal-stability. Notably, an interesting reversible sponge-like water de-/ad-sorption behavior of **1** during the thermal re-/de-hydration processes, associated with colour-changing property and solid-state structural transformation between its de-hydrated and re-hydrated forms, is observed; this was successfully identified by cyclic TG analysis, and further demonstrated by PXRD measurements and morphological study. These thermally-induced, breathing-type dynamic effects suggest a reversible solid-state structural transformation of the entangled structure of **1** after water removal and retrieval from the pores, which is in agreement with the water-sorption studies, and could be attributed to the significant intra-/inter-CPs hydrogen bonding interactions and inter-CPs π–π stacking interaction. This may be developed as a potential application of a thermally-induced moisture sensor. In this work, we successfully obtained a unique 3D-entangled supramolecular compound which displays interesting structural characteristics: reversible thermal-induced de-/re-hydration structural transformation associated with colour-changing behavior, magnetic property, and water hysteresis phenomenon in water vapor ad-/de-sorption isotherms. We thus believe that this nonporous composite entangled network should spark a broad spectrum of interest in the field of supramolecular chemistry.

## Figures and Tables

**Table 1:** Crystal data and refinement details of compound **1**.

Empirical Formula	C_75_H_82_N_8_Ni_4_O_30_	Formula Mass (g mol^−1^)	1810.33
crystal system	Orthorhombic	space group	*P*na2_1_
*a*/Å	20.3669(9)	α (°)	90
*b*/Å	13.6680(6)	β (°)	90
*c*/Å	27.1571(12)	γ (°)	90
*V*/Å^3^	7559.9(6)	Z	4
*D*_calcd_ (g cm^−3^)	1.591	θ range (deg.)	1.49–27.50
μ/mm^–1^	1.075	*T* (K)	150(2)
total no. of data collected	56,835	no. of unique data	13,812
*R*_1_, *wR*_2_^1^ (I > 2σ(I))	0.0636, 0.1226	*R*_1_, *wR*_2_^1^ (all data)	0.0856, 0.1324
GOF ^2^	1.072	refine params	1050

^1^*R*_1_ = ∑||*F*_o_ − *F*_c_||/∑|*F*_o_|; *wR*_2_(*F*^2^) = [∑w|*F*_o_^2^ − *F*_c_^2^|^2^/∑w(*F*_o_^4^)]^1/2^; ^2^ GOF = {∑[w|*F*_o_^2^ − *F*_c_^2^|^2^]/(*n* − *p*)}^1/2^.

## Supplementary Materials

The following are available online at http://www.mdpi.com/2073-4360/10/9/1014/s1, Figure S1: The coordination environments of the Ni(II) ions in (a) [Ni(dpe)(Hbtc)(H_2_O)], **A**; (b) [Ni(dpe)(btc)(H_2_O)]^−^, **B**; (c) [Ni(dpe)(btc)(H_2_O)_3_]^−^, **C**; (d) [Ni(dpe)(H_2_O)_4_]^2+^, **D**. ORTEP drawing with 30% thermal ellipsoids. The solvated water molecules and H atoms are omitted for clarity, Table S1: Bond lengths (Å) around Ni(II) ions in **1**, Table S2: The related parameters of O–H…O hydrogen bonds for **1**, Table S3: π–π interactions (face-to-face) in **1**.
