# Supplementary material for: Sponge-Like Water De-/Ad-Sorption versus Solid-State Structural Transformation and Colour-Changing Behavior of an Entangled 3D Composite Supramolecuar Architecture, [Ni4(dpe)4(btc)2(Hbtc)(H2O)9]·3H2O
Source: Polymers (Basel). 2018 Sep 11;10(9):1014. doi: 10.3390/polym10091014 (PMC6403924; doi:10.3390/polym10091014)
Supplement: Supplementary file 1 [file polymers-10-01014-s001.pdf]

Supplementary Information

Sponge-like Water de-/ad-sorption versus Solid-state Structural Transformation and Colour-Changing behavior of An Entangled 3D Composite Supramolecuar Architecture, $[Ni_4(dpe)_4(btc)_2(Hbtc)(H_2O)_9]\cdot 3H_2O$

Chih-Chieh Wang [1,*], Szu-Yu Ke [1], Kuan-Ting Chen [1], Ning-Kuei Sun [1], Wei-Fung Liu [1], Mei-Lin Ho [1,*], Bing-Jyun Lu [1], Yi-Ting Hsieh [1], Yu-Chun Chuang [2], Gene-Hsiang Lee [3], Shi-Yi Huang [4], and En-Che Yang [4,*]

[1] Department of Chemistry, Soochow University, Taipei 11102, Taiwan;
[2] National Synchrotron Radiation Research Center, Hsinchu 30076, Taiwan;
[3] Instrumentation Center, National Taiwan University, Taipei 10617
[4] Department of Chemistry, Fu Jen Catholic University, Taiwan.

Supporting Information

Table S1. Bond lengths (Å) around Ni(II) ions in **1**.

Table S2. The related parameters of O–H···O hydrogen bonds for **1**[a]

Table S3. π–π interactions (face-to-face) in **1**[a]

Figure S1 The coordination environments of the Ni(II) ions in (a) $[Ni(dpe)(Hbtc)(H_2O)]$, **A**; (b) $[Ni(dpe)(btc)(H_2O)]^-$, **B**; (c) $[Ni(dpe)(btc)(H_2O)_3]^-$, **C**; (d) $[Ni(dpe)(H_2O)_4]^{2+}$, **D**. ORTEP drawing with 30 % thermal ellipsoids. The solvated water molecules and H atoms are omitted for clarity.

Table S1. Bond Lengths (Å) around Ni(II) ions in **1**[a]

**CP A**			
Ni(1)–O(1)	2.028(4)	Ni(1)–O(7)	2.055(4)
Ni(1)–O(3)$_i$	2.124(4)	Ni(1)–O(4)$_i$	2.124(4)
Ni(1)–N(1)	2.139(5)	Ni(1)–N(2)	2.091(5)
**CP B**			
Ni(3)–O(15)	2.026(4)	Ni(3)–O(21)	2.068(3)
Ni(3)–O(23)	2.086(4)	Ni(3)–O(22)	2.087(4)
Ni(3)–N(5)	2.120(5)	Ni(3)–N(6)	2.140(5)
**CP C**			
Ni(2)–O(8)	2.031(4)	Ni(2)–O(14)	2.055(4)
Ni(2)–O(10)$_{ii}$	2.108(4)	Ni(2)–O(11)$_{ii}$	2.110(4)
Ni(2)–N(4)	2.123(5)	Ni(2)–N(3)	2.143(4)
**CP D**			
Ni(4)–O(24)	2.035(4)	Ni(4)–O(26)	2.053(4)
Ni(4)–O(25)	2.079(4)	Ni(4)–O(27)	2.083(4)
Ni(4)–N(8)	2.092(6)	Ni(4)–N(7)	2.104(5)

[a] Symmetry transformations used to generate equivalent atoms : i = x−1/2, −y+1/2, z; ii = x-1/2, -y+3/2, z.

Table S2. The related parameters of O−H···O hydrogen bonds for **1**[a]

D−H ···A	D−H (Å)	H ···A (Å)	D ···A (Å)	∠ D−H ···A (°)
O(7)−H(7A)···O(20)	0.85	1.955	2.786	165.7
O(7)−H(7B)···O(2)	0.85	1.891	2.664	151.0
O(14)−H(14A)···O(20)$_{i}$	0.83	1.959	2.708	150.0
O(14)−H(14B)···O(9)	0.85	1.765	2.609	169.3
O(21)−H(21A)···O(17)$_{ii}$	0.82	1.929	2.747	171.9
O(22)−H(22A)···O(16)	0.86	1.666	2.518	172.1
O(22)−H(22B)···O(18)$_{ii}$	0.86	1.738	2.591	170.2
O(23)−H(23B)···O(9)$_{iii}$	0.85	1.996	2.773	151.6
O(24)−H(24A)···O(12)	0.87	1.836	2.660	156.9
O(25)−H(25A)···O(19)	0.87	1.861	2.682	157.3
O(25)−H(25B)···O(26)	0.84	2.249	2.861	129.7
O(26)−H(26A)···O(18)	0.84	2.074	2.830	149.2
O(26)−H(26B)···O(13)	0.85	1.744	2.581	166.5
O(27)−H(27B)···O(3)	0.87	1.886	2.753	173.4
O(21)−H(21B)···O(29)	0.83	2.063	2.813	149.7
O(23)−H(23A)···O(29)	0.84	1.826	2.643	163.7
O(24)−H(24B)···O(28)$_{iv}$	0.87	1.925	2.723	152.2
O(27)−H(27A)···O(30)	0.86	2.005	2.783	150.3
O(28)−H(28A)···O(30)$_{ii}$	0.85	1.963	2.796	166.7
O(28)−H(28B)···O(17)	0.85	1.896	2.736	169.0
O(29)−H(29A)···O(10)$_{iii}$	0.85	2.373	2.750	107.4
O(29)−H(29B)···O(19)$_{v}$	0.86	2.230	2.777	121.4
O(30)−H(30B)···O(20)$_{v}$	0.89	2.197	2.941	141.0
O(5)−H(5)···O(22)$_{vi}$	0.84	1.742	2.580	175.6

[a] Symmetry code : i = ½−x, ½+y, −½+z ; ii = −½+x, ½−y, z ; iii = ½−x, −½+y, ½+z ; iv = ½+x, 3⁄2−y, z ; v = −½+x, ½−y, z ; vi = ½−x, −½+y, −½+z.

Table S3. π–π interactions (face-to-face) in **1**[a]

Ring(i) → Ring(j)	Slip angle[b] (i,j)/°	Interplanar (i,j) distance[c]/Å	Horizontal shift between the (i,j) ring centroids[d]/Å	Distance between the (i,j) ring centroids/Å
R(1)→R(2)	16.3(4)	3.358(7)	0.979(7)	3.498(7)
R(2)→R(3)	34.4(4)	3.295(7)	2.259(7)	3.995(7)
R(3)→R(4)$_i$	22.0(4)	3.324(7)	1.340(7)	3.584(7)
R(5)→R(6)$_i$	22.5(4)	3.441(7)	1.424(7)	3.724(7)

[a] Symmetry code: i = ½–x, ½+y, –½+z; R(1) = C(1)–C(2)–C(3)–C(4)–C(5)–C(6); R(2) = N(7)–C(64)–C(65)–C(66)–C(67)–C(68); R(3) = C(22)–C(23)–C(24)–C(25)–C(26)–C(27); R(4) = N(6)–C(59)–C(60)–C(61)–C(62)–C(63); R(5) = N(3)–C(31)–C(32)–C(33)–C(34)–C(35); R(6) = C(43)–C(44)–C(45)–C(46)–C(47)–C(48).

[b] Slip angle: the angle formed between the ring-centroid vector (CC) and the ring normal to one of the benzene or pyridine planes.

[c] Interplanar distance: the perpendicular distance between two parallel rings.

[d] Horizontal shift between the ring centroids: a shift from the face-to-face alignment.

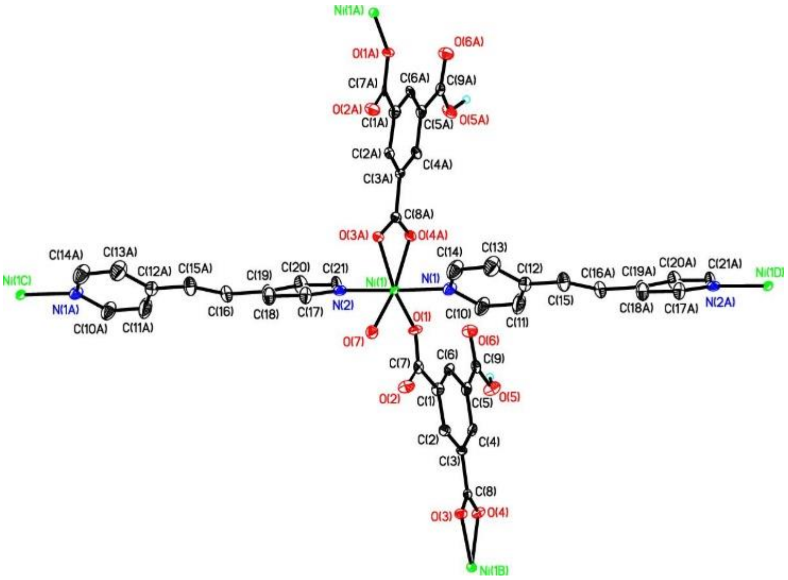

Ni(1A)
O(1A)
O(6A)
C(7A)
C(6A)
C(9A)
O(2A)
C(1A)
C(5A)
O(5A)
C(2A)
C(4A)
C(3A)
C(8A)
O(3A)
O(4A)
C(14A)
C(13A)
C(12A)
C(15A)
C(19)
C(20)
C(21)
Ni(1)
N(1)
C(14)
C(13)
C(12)
C(16A)
C(19A)
C(20A)
C(21A)
Ni(1C)
N(1A)
C(16)
C(18)
C(17)
N(2)
C(10)
C(11)
C(15)
C(18A)
C(17A)
N(2A)
Ni(1D)
C(10A)
C(11A)
O(7)
O(1)
O(6)
C(7)
C(6)
C(9)
O(2)
C(1)
C(5)
O(5)
C(2)
C(4)
C(3)
C(8)
O(3)
O(4)
Ni(1B)

(a)

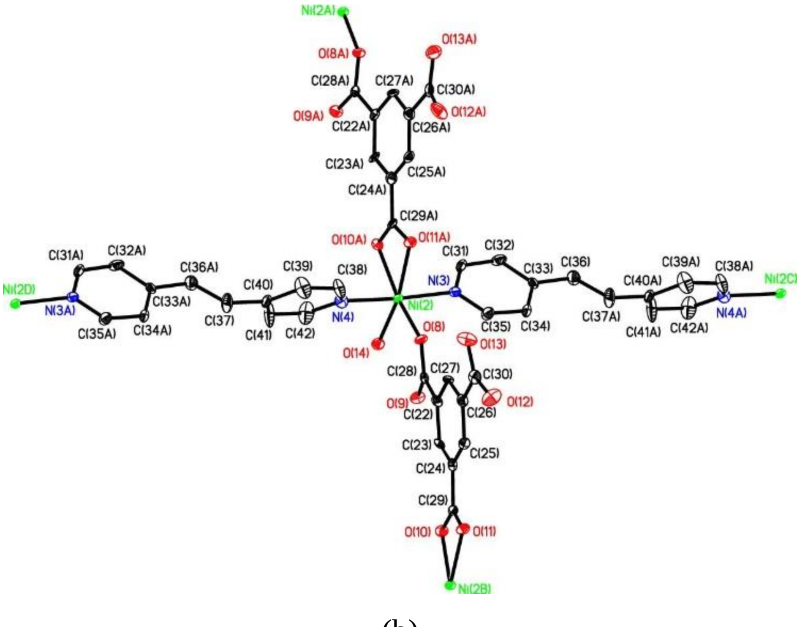

Ni(2A)
O(8A)
O(13A)
C(28A)
C(27A)
C(30A)
O(9A)
O(12A)
C(22A)
C(26A)
C(23A)
C(25A)
C(24A)
C(29A)
O(10A)
O(11A)
C(31A)
C(32A)
C(36A)
C(39)
C(38)
C(31)
C(32)
C(33)
C(36)
C(39A)
C(38A)
Ni(2D)
N(3A)
C(33A)
C(40)
N(3)
Ni(2)
C(40A)
N(4A)
Ni(2C)
C(35A)
C(34A)
C(37)
C(41)
C(42)
N(4)
C(35)
C(34)
C(37A)
C(41A)
C(42A)
O(14)
O(8)
O(13)
C(28)
C(27)
C(30)
O(9)
C(22)
C(26)
O(12)
C(23)
C(25)
C(24)
C(29)
O(10)
O(11)
Ni(2B)

(b)

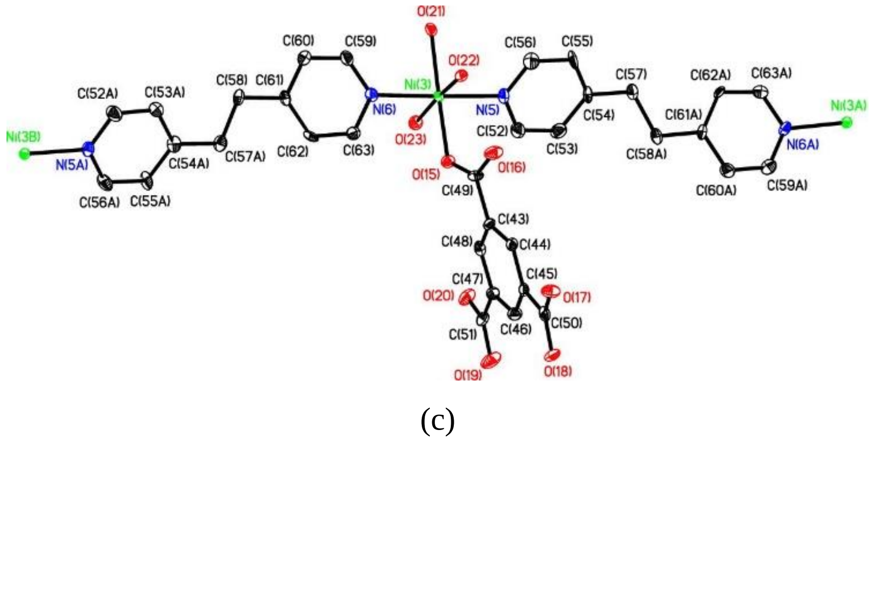

O(21)
C(60)
C(59)
O(22)
C(56)
C(55)
C(52A)
C(53A)
C(58)
C(61)
Ni(3)
C(57)
C(62A)
C(63A)
N(6)
N(5)
C(54)
C(61A)
Ni(3A)
Ni(3B)
O(23)
C(52)
N(6A)
N(5A)
C(54A)
C(57A)
C(62)
C(63)
C(53)
C(58A)
C(56A)
C(55A)
O(15)
O(16)
C(49)
C(60A)
C(59A)
C(43)
C(48)
C(44)
C(47)
C(45)
O(20)
O(17)
C(51)
C(46)
C(50)
O(19)
O(18)

(c)

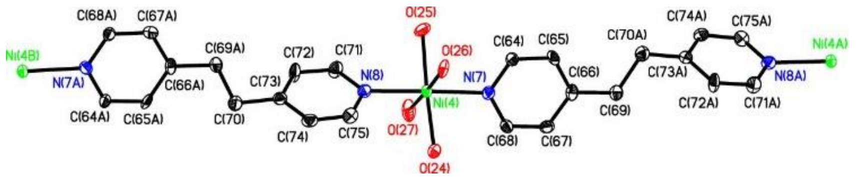
C(68A)
C(67A)
Ni(4B)
N(7A)
C(66A)
C(69A)
C(72)
C(71)
C(73)
N(8)
O(25)
O(26)
N(7)
C(64)
C(65)
C(66)
C(70A)
C(74A)
C(75A)
Ni(4A)
C(73A)
N(8A)
Ni(4)
O(27)
C(64A)
C(65A)
C(70)
C(74)
C(75)
O(24)
C(68)
C(67)
C(69)
C(72A)
C(71A)

(d)

Figure 1
